# Synaptic and cellular organization of layer 1 of the developing rat somatosensory cortex

**DOI:** 10.3389/fnana.2013.00052

**Published:** 2014-01-16

**Authors:** Shruti Muralidhar, Yun Wang, Henry Markram

**Affiliations:** ^1^Laboratory of Neural Microcircuitry, Brain Mind Institute, École Polytechnique Fédérale de LausanneLausanne, Switzerland; ^2^Key Laboratory of Visual Science and National Ministry of Health, School of Optometry and Opthalmology, Wenzhou Medical CollegeWenzhou, China; ^3^Caritas St. Elizabeth's Medical Center, Genesys Research Institute, Tufts UniversityBoston, MA, USA

**Keywords:** neocortical circuits, layer 1, interneurons, inhibition, gaba receptors

## Abstract

Layer 1 of the neocortex is sparsely populated with neurons and heavily innervated by fibers from lower layers and proximal and distal brain regions. Understanding the potential functions of this layer requires a comprehensive understanding of its cellular and synaptic organization. We therefore performed a quantitative study of the microcircuitry of neocortical layer 1 (L1) in the somatosensory cortex in juvenile rats (P13–P16) using multi-neuron patch-clamp and 3D morphology reconstructions. Expert-based subjective classification of the morphologies of the recorded L1 neurons suggest 6 morphological classes: (1) the Neurogliaform cells with dense axonal arborizations (NGC-DA) and with sparse arborizations (NGC-SA), (2) the Horizontal Axon Cell (HAC), (3) those with descending axonal collaterals (DAC), (4) the large axon cell (LAC), and (5) the small axon cell (SAC). Objective, supervised and unsupervised cluster analyses confirmed DAC, HAC, LAC and NGC as distinct morphological classes. The neurons were also classified into 5 electrophysiological types based on the Petilla convention; classical non-adapting (cNAC), burst non-adapting (bNAC), classical adapting (cAC), classical stuttering (cSTUT), and classical irregular spiking (cIR). The most common electrophysiological type of neuron was the cNAC type (40%) and the most common morpho-electrical type was the NGC-DA—cNAC. Paired patch-clamp recordings revealed that the neurons were connected via GABAergic inhibitory synaptic connections with a 7.9% connection probability and via gap junctions with a 5.2% connection probability. Most synaptic connections were mediated by both GABA_A_ and GABA_B_ receptors (62.6%). A smaller fraction of synaptic connections were mediated exclusively by GABA_A_ (15.4%) or GABA_B_ (21.8%) receptors. Morphological 3D reconstruction of synaptic connected pairs of L1 neurons revealed multi-synapse connections with an average of 9 putative synapses per connection. These putative synapses were widely distributed with 39% on somata and 61% on dendrites. We also discuss the functional implications of this L1 cellular and synaptic organization in neocortical information processing.

## Introduction

Layer 1 (L1) of the neocortex is located immediately below the *pia mater* and consists of a low density of neurons and glia along with apical dendrites of pyramidal cells of the underlying layers, and axon collaterals other neocortical areas and the thalamus (Marin-Padilla and Marin-Padilla, 1982). L1 is known to be important in neocortical development, but its role in neocortical function is still not clear. One of the first reports, by Somogyi et al. found the density of L1 neurons in the cat visual cortex to be 1173 neurons/mm^3^ (Gabbott and Somogyi, 1986) as compared to over 100,000/mm^3^ for the deeper layers (data not shown). L1 cells are inhibitory neurons, containing the neurotransmitter γ-amino butyric acid, (GABA) and they make up 8.3% of the total GABAergic cells in the neocortex. The earliest cell type observed and defined by Ramon y Cajal and Magnus Gustaf Retzius (Cajal, 1995) was the Cajal Retzius (CR) cell. It is one of the first to form during development (Marin-Padilla, 1990; Marín-Padilla, 1999) and is critical for cortical lamination due to the expression of Reelin (Marin-Padilla and Marin-Padilla, 1982; Río et al., 1997; Marín-Padilla, 1998). However, they are only transiently expressed and are no longer present by post-natal day 11 (P11) in rodent cortex (Portera-Cailliau et al., 2005).

A wide variety of other types of L1 neurons however persist in L1. Five morphological types were described based on Golgi and Nissl staining in the developing occipital cortex of rats (P2 to P35). These were classified as fetal horizontal cells, persisting horizontal cells, vertical cells, classical non-pyramidal cells and non-axonal cells (Bradford et al., 1977). Four morphological types were reported in the juvenile cortex of rats (P0–P21) classified as Cajal-Retzius cells, Cells with confined axons, Cells with Axons not confined to L1 and Vertical Axon cells (Zhou and Hablitz, 1996). Along with classification by morphological properties, Hestrin and Armstrong first reported electrophysiological properties of L1 cells found in three morphological classes of neuron in the somatosensory cortex (SSC) of the rat (P7 to P19); Axon Horizontal (AHC), Axon Descending (ADC) and Neurogliaform (Ngf) cells (Hestrin and Armstrong, 1996). More recent data comes from a study by Williams et al. (Wozny and Williams, 2011) where the L1 cells from the adult rat SSC (P24–P36) were classified into 3 major morphological classes. These include Ngf cells (NGFC), cells with vertical axons, and Chandelier-like cells. Three major electrophysiological types were reported including Regular spiking (RS), Fast spiking (FS), Burst spiking (BS) and classical accommodating (cAC). Ward's classification and the Petilla convention were combined and used to give 4 groups—NGFC-cAC, cAC-FS, cAC-BS, and NGFC-cAC (Ward, 1963; Ascoli et al., 2008). Bekkers et al. documented the constituent cells of L1 of the rat anterior piriform cortex using GAD67-GFP (Glutamate decarboxylase -67—Green Fluorescent Protein) expressing transgenic mice (Suzuki and Bekkers, 2010a,b). They found 7 different types of morpho-electrical classes in mice, between the ages of P14–P25. Synaptic connections formed between L1 cells are largely unknown with the exception of two studies that document the synaptic connections between L1 cells. Cruikshank et al. have observed IPSPs between L1 cells to have slow kinetics and strong short term depression (Cruikshank et al., 2012). Chu et al. have described inhibitory synaptic connections between Late and Non-Late Spiking cells as mediated by classical and non-classical GABA_A_ receptors (Chu et al., 2003).

Taken together, these studies do not provide a consistent view of the morphological and electrical cell types in L1 or the synaptic connections they form among each other. We therefore have endeavored to establish a consistent and reproducible classification scheme for L1 neurons. All the experiments were performed on the developing rat neocortex (post-natal days 13–16) using single and multi-cell patch-clamp electrophysiology, pharmacological manipulations followed by histochemical staining and 3D morphological reconstructions. We recorded the electrophysiological properties of 810 L1 neurons, labeled 158 with biocytin, and 3D reconstructed 95 of these neurons. We recorded the synaptic physiology of 142 synaptic connections, labeled 18, and 3D reconstructed 4 exemplars representative of each type of synaptic connection. Morphological cell types were classified using subjective and objective classification based on axonal and dendritic features. Electrophysiological cell types where classified according to the Petilla convention (Ascoli et al., 2008) and based on response properties to step depolarization. Along with detecting and quantifying gap junctions, synaptic connections were classified according whether they showed properties of GABA_A_, GABA_B_ or GABA_A_ + GABA_B_.

## Methods

### Slice experiments

Experiments were carried out according to Swiss National and Institutional guidelines. 13–16 day old, non-anaesthetized Wistar rats were rapidly decapitated and their brains carefully removed and kept in iced, artificial cerebrospinal fluid (aCSF). 300 μm thick parasaggital slices, approximately 1.7–2.2 mm lateral to the midline slices were cut on an HR2 vibratome (Sigmann Elektronik, Heidelberg, Germany) with a 5° incline. The primary SSC on the slice was located by the anterior extremity of the hippocampus (bend of the CA3 region). Hind limb SSC was designated as ±1 mm from this extremity and all cells in L1 were patched within this region. The slices were incubated at 35°C for 30 min and left at room temperature in the holding chamber, until recording.

### Electrophysiology and analyses

Cells were visualized by Infrared Differential Interference Contrast microscopy (IR-DIC) (Olympus BX51WI microscope, PCO CCD imaging or VX55 Photonics camera) (Stuart et al., 1993). L1 cells were selected according to their positions (not more than 100 microns away from the pia). Cells at the layer1—layer 2–3 interface were avoided. Slices kept on the electrophysiological setup were continuously superfused with aCSF containing (in mM) 125 NaCl, 25 NaHCO_3_, 2.5 KCl, 1.25 NaH_2_PO_4_, 2 CaCl_2_, 1 MgCl_2_, and 25 D-glucose, bubbled with 95% O_2_–5% CO_2_. The intracellular pipette solution contained (in mM) 110 Potassium Gluconate, 10 KCl, 4 ATP-Mg, 10 Phosphocreatine, 0.3 GTP, 10 HEPES and 13 Biocytin, adjusted to 290–300 mOsm/Lt with D-Mannitol (25–35 mM) at pH 7.3. Chemicals were sourced from from Sigma Aldrich (Stenheim, Germany) or Merck (Darmstadt, Germany). Gabazine (SR 95331 hydrobromide Ref. no. 1262, Tocris Biosciences) was used at a working concentration of 20 μ M, diluted from a 20 mM stock solution and CGP55845 (Tocris Biosciences, Ref. no. 1248) was used at working concentration of 4 μ M, diluted from a 10 mM stock made in DMSO. Care was taken to keep the working concentration of DMSO to levels less than 1 in 1000 to avoid any chances of cellular toxicity.

Multiple somatic whole cell recordings (1–6 cells) were performed with Axopatch 200B amplifiers in current clamp mode at 34 ± 1°C bath temperature. Data acquisition was performed via an ITC-18, connected to a Macintosh, running a custom written routine in IGOR Pro (Version 6.05A, Wavemetrics, Portland, OR, USA). Voltage signals were sampled at rates between 5 and 10 kHz and filtered with a 2 kHz Bessel filter. Patch pipettes with a tip resistance of 3–8 MΩ were pulled with a Flaming/Brown micropipette puller P-97 (Sutter Instruments and Co.) using borosilicate glass capillaries with filaments (Article code number 1403513, Hilgenberg). Experiments were performed both with standard intracellular solution (as described in Slice Preparation) and with Amphotericin B for normal and perforated patch-clamp, respectively.

Intrinsic electrical properties of the cells were measured using different stimuli and calibrated current intensities. Calibration was performed by varying the amplitude of a square pulse of 50 pA until it elicited a single action potential. The cells were then left undisturbed at native resting membrane potentials. As soon as electrical access was obtained, a pre-defined set of stimuli (e-code) was applied.

Cells were classified in terms of the basic types defined in the Petilla Interneuron Convention (Ascoli et al., 2008). We identified five electrophysiological neuron types (e-types), each characterized by a specific firing pattern: classical Accommodating Cells (cAC), classical Non-Accommodating Cells (cNAC), bursting Non-Accommodating Cells (bNAC), classical Stuttering Cells (cSTUT) and classical Irregular Spiking Cells (cIR). For each cell, we plotted Inter Spike Intervals (ISIs) against AP sequence order. This gave us a linear regression line and a coefficient of correlation to quantify the goodness of fit to the linear regression. Cells whose regression line had a slope greater than or equal to 1 and whose sum of the Root Mean Square (RMS) error was less than 30 (mean = 28.76), were classified as Accommodating Cells. Cells whose regression line had a slope less than 1 and whose sum of the RMS errors were less than 30 (mean = 20.11), were classified as Non-Accommodating Cells. Cells whose regression lines had RMS errors greater than 30 were separated into cSTUT/cIR group for further anaylsis. For our final classification, we combined this information with information about the pattern of ISI in the AP train. We also further investigated the properties of cells using other stimuli as listed in Table 3.

Synaptic connections were measured by applying pre-synaptic stimuli (a train of action potentials (APs) elicited by current pulses at varying frequencies) plus a “recovery test pulse” (RTR) 500 ms after the train. The pulse duration was 3 ms with amplitude of 1–2 nA. This was usually sufficient to trigger reliable and precisely timed APs. The post-synaptic membrane potential was current clamped to approximately −57 mV in order to increase the electric driving force (*E_Cl_* = −69 mV). If a synaptic connection was detected, we iterated the procedure 10 to 20 times with inter-stimulus intervals (ISIs) of 30, 60, 90, and 120 s. C.V (Co-efficient of Variation) was calculated by dividing the SD (Standard Deviation) of the single IPSP amplitudes by the mean value.

Single cell and synaptic parameters were extracted with the help of custom-made scripts in IGOR Pro (Version 6.05A) and Matlab (R2009b). Total amplitude values of the Inhibitory Post-Synaptic Potentials (IPSPs) were calculated peak to peak. Rise and decay times of the IPSPs were calculated at 20–80% of the total amplitude.

### Perforated patch recording

A 200 μg/ml stock solution of Amphotericin B (Sigma-Aldrich—Cat no. A-4888) in Dimethylsulfoxide (DMSO) was prepared. A working concentration of 100 μg/ml was achieved by sonicating 8 μ l of stock in 1 ml of Intracellular Solution (ICS). The tip of the pipette was back-filled with normal ICS solution until the end of the taper. This was then layered with approximately 20 μ l of Amphotericin-ICS following which; the pipette was quickly fitted on to the silver wire electrode. Care was taken to apply minimum positive pressure and to minimize movement within the tissue before reaching the chosen cells. Once adequate electrical access (access resistance values <20 MΩ) was achieved, the cell was current clamped and stimulation protocols applied.

### Morphological reconstructions

The staining and mounting procedure resulted in shrinkage of the slice to 50–75% of its original 300 μm thickness. Reconstructions were corrected for this value. The anisotropic shrinkage along the X–Y plane was around 0–10% and not corrected. The cells were reconstructed in 3D under an Olympus BX 51W microscope with a water-immersion 60 (NA 0.9) or an oil-immersion 100X (NA 1.35) objective using Neurolucida software (MicroBrightField, Magdeburg, Germany). Reconstructed neurons and connections were analyzed both with NeuroExplorer (MicroBrightField) and custom written scripts in Matlab. The list of parameters extracted closely match those reported in (Wang et al., 2002). Putative synaptic contacts were identified as close appositions of boutons and dendrites in the same focal plane.

### Morphology and analyses

#### Subjective morphological analyses

Morphological parameters (similar to those listed in (Wang et al., 2004) were extracted using NeuroExplorer and used as the basis for an initial subjective classification of morphological types. Table 1 lists the main parameters used in the classification and the *p*-values that are most significant.

**Table 1:** **Summary of the expert chosen morphometric parameters of soma, dendrites and axon, of the 6 groups of L1 cells, with their corresponding subgroups**.

		**NGC-DA**	**NGC-SA**	***P***	**HAC**	**DAC**	***P***	**LAC**	**SAC**	***P***
		***n* = 17 (15%)**	***n* = 16 (14%)**		***n* = 19 (17%)**	***n* = 16 (14%)**		***n* = 11 (10%)**	***n* = 14 (13%)**	
**SOMA**										
	Perimeter (μm)	48 ± 1	49 ± 2	0.56	49 ± 1	51 ± 2	0.34	47 ± 1	43 ± 2	0.10
	Area (μm^2^)	172 ± 11	178 ± 13	0.68	174 ± 6	191 ± 14	0.27	162 ± 9	135 ± 12	0.07
**DENDRITE**										
	Max horizontal extend (μm)	122 ± 9	191 ± 17	0.00^**^	227 ± 14	305 ± 48	0.12	211 ± 18	218 ± 26	0.82
	Max vertical extend (μm)	133 ± 12	150 ± 14	0.36	170 ± 14	209 ± 16	0.06	187 ± 17	194 ± 14	0.77
	H/V	1.0 ± 0.1	1.4 ± 0.1	0.01^**^	1.4 ± 0.1	1.5 ± 0.2	0.80	1.2 ± 0.1	1.2 ± 0.1	0.91
	Dendrite number	5 ± 0.4	6 ± 0.5	0.38	5 ± 0.4	6 ± 0.5	0.38	6 ± 0.5	5 ± 0.4	0.03^*^
	Length (μm)	1470 ± 239	1821 ± 147	0.19	1808 ± 139	2492 ± 336	0.07	1985 ± 160	1636 ± 149	0.04^*^
	Tree length (μm)	280 ± 32	336 ± 27	0.16	363 ± 31	479 ± 76	0.16	337 ± 40	322 ± 29	0.76
	Segment length (μm)	26 ± 2	33 ± 2	0.01^**^	45 ± 3	50 ± 4	0.27	34 ± 1	38 ± 3	0.18
	Segment number	58 ± 9	56 ± 4	0.83	43 ± 4	53 ± 7	0.20	60 ± 6	43 ± 5	0.02^*^
	Tortuosity	1.28 ± 0.02	1.24 ± 0.01	0.08	1.29 ± 0.02	1.25 ± 0.01	0.08	1.26 ± 0.01	1.18 ± 0.01	0.00^**^
	Average order	4 ± 0.2	4 ± 0.2	0.40	4 ± 0.2	4 ± 0.3	0.86	4 ± 0.3	4 ± 0.2	0.66
	Maximum order	7 ± 0.3	7 ± 0.3	1.00	6 ± 0.5	7 ± 0.7	0 76	7 ± 0.5	6 ± 0.3	0.18
	Maximum angle (deg)	68 ± 2	66 ± 2	0.34	64 ± 1	65 ± 2	0.67	63 ± 2	62 ± 3	0.83
	Planar angle (deg)	51 ± 1	48 ± 1	0.08	46 ± 2	48 ± 1	0.39	47 ± 1	46 ± 2	0.55
	Local angle (deg)	59 ± 1	56 ± 1	0.05	60 ± 1	59 ± 1	0.50	56 ± 1	57 ± 2	0.63
	Local spline angle (deg)	53 ± 1	50 ± 1	0.02	53 ± 1	53 ± 1	0.69	51 ± 1	49 ± 2	0.32
**AXON**										
	Max horizontal extent (H) (urn)	481 ± 35	513 ± 33	0.50	826 ± 47	1035 ± 63	0.01^**^	879 ± 108	411 ± 37	0.00^**^
	Max vertical extent(V) (μm)	263 ± 33	163 ± 10	0.01^**^	202 ± 16	602 ± 49	0.00^**^	316 ± 29	251 ± 40	0.18
	H/V	2.0 ± 0.2	3.3 ± 0.3	0.00^**^	4.5 ± 0.4	2.0 ± 0.3	0.00^**^	3.0 ± 0.4	2.0 ± 0.2	0.04^*^
	Tree length (μm)	11067 ± 1475	6134 ± 655	0.00^**^	9688 ± 1099	14584 ± 2084	0.04^**^	15082 ± 1084	3325 ± 543	0.00^**^
	Segment length (μm)	42 ± 2	45 ± 2	0.38	67 ± 3	60 ± 3	0.15	44 ± 2	45 ± 2	0.79
	Segment number	270 ± 38	142 ± 16	0.00^**^	155 ± 21	273 ± 46	0.03^*^	342 ± 26	89 ± 17	0.00^**^
	Tortuosity	1.30 ± 0.01	1.29 ± 0.01	0.49	1.27 ± 0.01	1.24 ± 0.01	0.08	1.31 ± 0.03	1.22 ± 0.02	0.01^**^
	Maximum order	16 ± 1	15 ± 1	0 24	14 ± 1	20 ± 2	0.01^**^	21 ± 2	12 ± 1	0.00^**^
	Maximum angle (deg)	73 ± 1	74 ± 2	0.65	74 ± 2	75 ± 1	0.72	72 ± 2	78 ± 1	0.02^*^
	Planar angle (deg)	53 ± 1	53 ± 1	0.53	52 ± 1	52 ± 1	0.79	50 ± 1	55 ± 1	0.01^**^
	Local angle (deg)	58 ± 1	60 ± 1	0.31	61 ± 1	62 ± 1	0 64	59 ± 1	61 ± 1	0.47
	Local spline angle (deg)	52 ± 1	54 ± 1	0.23	55 ± 1	55 ± 1	0.69	54 ± 1	55 ± 1	0.55
	Bouton density (number/μm)	0.20 ± 0.01	0.19 ± 0.01	0.52	0.20 ± 0.01	0.18 ± 0.01	0.17	0.20 ± 0.01	0.16 ± 0.01	0.03^*^

* *p ≤ 0.05*.

** *p ≤ 0.01*.

#### Objective morphological analyses

An objective analysis of the L1 morphologies was performed to validate the initial subjective classification scheme. Principal Component Analysis (PCA) and Linear Discriminant Analysis (LDA) were applied using custom Python code and the Scikit-learn machine learning Python module (Pedregosa et al., 2011) on the normalized dataset.

The central idea of PCA is to reduce the dimensionality of a dataset consisting of a large number of inter-related variables, while retaining as much as possible of the variation present in the dataset. The initial uncorrelated principal components (PCs) are transformed and ordered as per the amount of variation retained (Jolliffe, 2002).

LDA maximizes the ratio of between-class variance to within-class variance, enabling similar elements in the dataset to group together and maximizing the distance between dissimilar elements. In order to ensure that there was no overfitting, ten rounds of ten-fold cross-validation were performed on the same data set with class assignments randomized. A student's *t*-test was used to compare the scores from the actual data set to the pooled scores of the randomized data sets.

Additional descriptive morphological features were appended to the original list of parameters to better describe the spatial spread and the branching parameters of the reconstructions (Table 2).

**Table 2:** **A listing of all the morphometric features used for the objective analyses with PCA and LDA**.

	**Name**	**Variance**	**Var ratio**
1	Soma CSA	0.81	0.11
2	Basal number trunks	0.76	0.05
3	Basal mean trunk diameter	0.79	0.16
4	Basal number segments	0.72	0.07
5	Basal max branch order	0.73	0.02
6	Basal max path length	0.76	0.26
7	Basal max radial distance	0.76	0.30
8	Basal max degree	0.71	0.06
9	Basal total length	0.79	0.15
10	Basal total surface area	0.79	0.26
11	Basal total volume	0.74	0.28
12	Basal bif mean branch length	0.72	0.34
13	Basal horizontal range	0.75	0.29
14	Basal vertical range	0.81	0.21
15	Dendrite moment 1 (x)	0.73	0.03
16	Dendrite moment 1 (y)	0.75	0.03
17	Dendrite moment 1 (z)	0.76	0.09
18	Dendrite moment 2 (x)	0.72	0.27
19	Dendrite moment 2 (y)	0.75	0.24
20	Dendrite moment 2 (z)	0.77	0.26
21	Dendrite density	0.31	0.11
22	Bdend tortuosity	0.82	0.20
23	Hv_ratio_dend (raw h, w)	0.81	0.15
24	Axon mean trunk diameter	0.79	0.07
25	Axon number segments	0.80	0.38
26	Axon max branch order	0.73	0.24
27	Axon max path length	0.78	0.50
28	Axon max radial distance	0.80	0.48
29	Axon max degree	0.80	0.38
30	Axon total length	0.78	0.42
31	Axon total surface area	0.73	0.32
32	Axon total volume	0.64	0.16
33	Axon bif mean branch length	0.78	0.45
34	Axon horizontal range	0.82	0.59
35	Axon vertical range	0.76	0.73
36	Axon moment 1 (x)	0.76	0.06
37	Axon moment 1 (y)	0.75	0.11
38	Axon moment 1 (z)	0.83	0.13
39	Axon moment 2 (x)	0.83	0.61
40	Axon moment 2 (y)	0.72	0.62
41	Axon moment 2 (z)	0.80	0.33
42	Axon density	0.78	0.48
43	Axon tortuosity	0.79	0.18
44	H/V_ratio_axon (raw h, w)	0.77	0.52

*(CSA = Cross Section Area, bif = bifurcating)*.

**Table 3:** **Summary of the electrophysiological parameters of each of the firing pattern cell-types in L1**.

**E-parameters**	**Units**	**cNAD**		**cNAD (doublet)**		**cAD**		**bNAD**		**cSTUT**		**cIS**	
		**mean**	***SE***	**mean**	***SE***	**mean**	***SE***	**mean**	***SE***	**mean**	***SE***	**mean**	***SE***
AP1 amplitude	mV	60.85	8.02	66.74	5.78	63.88	5.79	60.47	5.34	64.58	5.95	66.94	6.32
AP1 duration	ms	2.43	0.34	2.53	0.39	2.53	0.73	2.57	0.42	2.66	0.66	2.37	0.58
AP1 half-width	ms	1.25	0.18	1.24	0.17	1.29	0.27	1.31	0.19	1.32	0.29	1.20	0.26
AP1 rise-time	ms	0.98	0.11	0.96	0.12	0.99	0.13	0.99	0.10	1.01	0.15	0.97	0.16
AP1 fall-time	ms	1.44	0.25	1.56	0.29	1.54	0.61	1.57	0.33	1.65	0.53	1.41	0.43
AP1 ahp-time	ms	2.99	1.97	1.29	0.68	2.77	2.10	2.19	1.82	2.36	1.14	1.98	1.83
AP1 rise-fate	mV/ms	63.02	12.42	70.23	8.89	65.75	9.91	61.43	7.89	65.93	12.25	70.75	11.43
AP1 fall-rate	mV/ms	43.78	10.39	44.07	8.69	45.45	13.17	40.02	8.84	42.75	11.63	50.64	12.98
AP1 fast AHP	mV	14.46	3.77	10.16	2.85	16.12	3.97	12.19	5.51	13.15	4.07	14.69	5.47
AP2 amplitude	mV	60.97	7.29	64.05	5.65	63.59	5.26	59.41	4.45	61.17	7.59	62.86	3.72
AP2 duration	ms	2.76	0.55	3.09	0.61	2.91	0.90	3.09	0.70	2.99	0.71	2.74	0.96
AP2 half-width	ms	1.45	0.26	1.55	0.26	1.53	0.41	1.62	0.34	1.55	0.35	1.46	0.51
AP2 rise-time	ms	1.06	0.14	1.06	0.14	1.07	0.17	1.09	0.13	1.08	0.18	1.06	0.27
AP2 fall time	ms	1.71	0.45	2.03	0.51	1.84	0.75	2.00	0.58	1.90	0.55	1.69	0.69
AP2 ahp-time	ms	1.41	1.45	1.14	1.31	1.56	1.57	2.71	1.76	2.09	1.26	2.06	1.23
AP2 rise-rate	mV/ms	58.91	11.64	61.56	8.95	60.93	11.71	55.39	8.75	58.54	14.19	62.68	15.50
AP2 fall-rate	mV/ms	37.98	10.27	33.39	8.63	39.45	14.56	32.27	9.99	34.90	10.61	42.18	14.80
AP2 fast AHP	mV	17.27	7.59	17.84	9.33	20.38	5.45	13.46	5.51	16.31	3.52	16.67	4.46
Input resistance for peak	MOhm	347.76	102.35	380.37	196.52	396.67	270.57	374.98	143.95	293.88	97.10	257.75	115.85
Input resistance for steady-state	MOhm	323.47	92.83	362.09	196.96	334.44	218.95	346.31	128.15	281.00	87.19	249.72	104.28
Time constant for delta pulse	ms	18.84	6.42	24.64	10.39	17.77	6.70	23.67	8.47	16.37	5.45	17.37	12.42
AP threshold	mV	−35.66	8.74	−38.40	3.32	−37.31	4.71	−37.01	6.49	−32.61	15.58	−39.60	3.21
sAHP maximum	mV	8.08	3.89	7.10	4.29	9.15	3.19	6.18	2.34	2.92	15.62	8.59	3.13
Minimum current to threshold	pA	41.82	31.51	43.71	24.55	35.44	29.49	37.52	17.13	38.62	14.83	42.77	25.59

A first LDA trial was performed with six groups, matching the initial subjective classification. The analysis was then re-run first removing the DAC group and then combining the NGC groups.

#### 3D visualization

Construction of large-scale 3D models of neural microcircuitry requires specialized applications to position and connect thousands of morphologically complex 3D neurons. The EPFL Blue Brain Project has developed custom software for this purpose. The BlueBuilder application allows a user to define the macroscopic shell and recipe for a neural microcircuit, load and position 3D neuron models within the shell, detect putative appositions between neurons, and ultimately export a circuit configuration file that other applications can use for simulation and visualization. In the work described here, 3D neural circuits were visualized using RTNeuron a multi-threaded multi-GPU (Graphics Processing Unit) application developed by the project (Hernando et al., 2008).

## Results

### Neuron morphologies in layer 1

#### Expert based subjective classification

**Figure 1:**
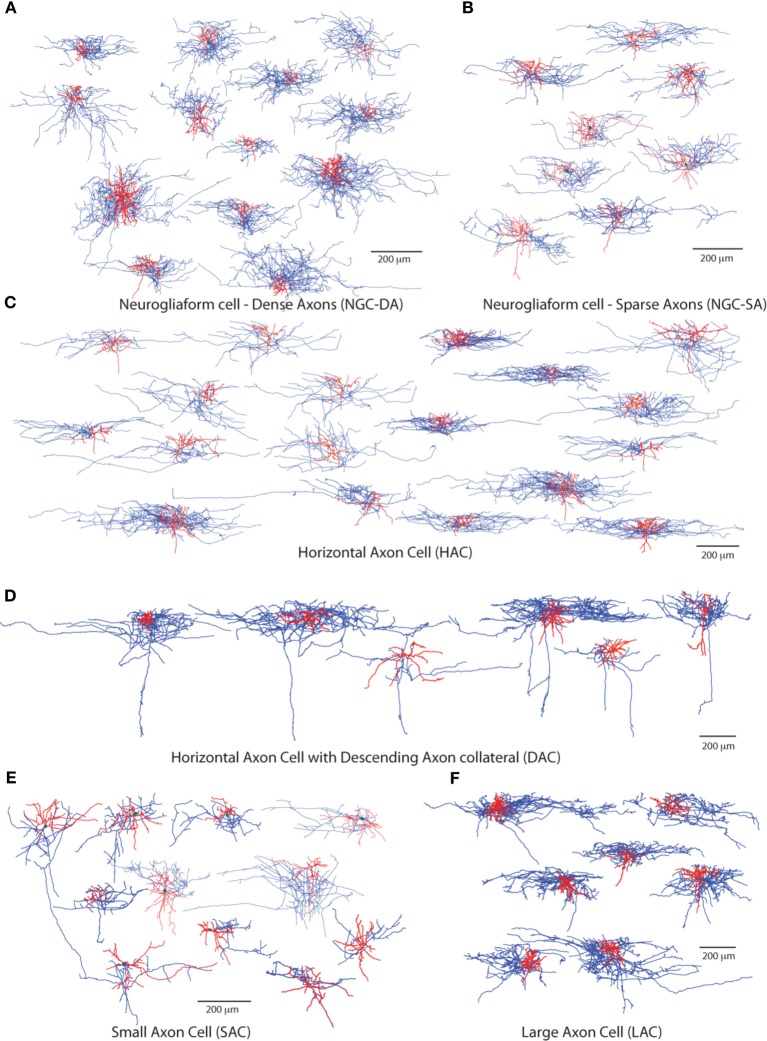
**Subjective Morphological types of cells found in L1**. The 6 major morphologically distinct groups of L1 cells. **(A)** Typical neurogliaform cells with dense axonal arbourisation (NGC-DA). **(B)** Atypical neurogliaform cells with sparse axonal arbourisation (NGC-SA). **(C)** Horizontal Axon cells (HAC) and **(D)** HACs with a descending axon (DAC) **(E)** dense and **(F)** small axon cells (LAC and SAC) Dendrites are shown in red and the axonal arbor in blue.

Out of a total of 158 stained L1 neurons, 95 were reconstructed in 3D for morphometric analysis and quantitative comparison. For each neuron we measured a set of dendritic and axonal features including segment length, segment tortuosity and branch angles (Figure 1). We produced an initial subjective classification of neurons based on key distinguishing morphometric features (Table 1): Ngf Cells with dense (NGC-DA) and sparse local axonal arborization (NGC-SA), Horizontal Axon Cells (HAC), Descending Axon Cell (DAC); Large Axon Cell (LAC) and Small axon cell (SAC). We also identified one neuron as a Cajal-Retzius cell.

***Neurogliaform cells with dense axonal arbors (NGC-DA)***. NGC-DA cells were very similar to the Ngf cells reported in other cortical layers showing small compact axonal arborization (Figure 1) (Kisvárday et al., 1990; Kawaguchi and Kubota, 1997; Szabadics et al., 2007). This confirmed by the values for horizontal and vertical axonal extents. (horizontal: 481 ± 35 μm and vertical: 263 ± 33 μm) (Table 1). These neurons also typically display the shortest dendritic segments (26 ± 2 μm).

***Neurogliaform cells with sparse axonal arbors (NGC-SA)***. Visually, NGC-SA neurons appeared as a sparser variation of NGC-DAs. They displayed similar axonal branching patterns in terms of segment length, tortuosity, and branch angles. However, NGC-SAs differed from NGC-DAs in that they displayed significantly smaller vertical arborizations with fewer and shorter axon collaterals (Table 1). On the other hand, the dendritic arborization was seen to extend further in the horizontally with longer dendritic segments with smaller branching angles (Table 1).

***Horizontal axon cells (HAC)***. HAC neurons were characterized by extensive horizontal axonal arborizations as compared to vertical extents, with long axonal segment lengths (67 ± 3 μm). As a result, their Horizontal/Vertical extent (H/V) ratio (H: 826 ± 47 μm, V: 202 ± 16 μm) was higher as compared to other cell types.

***Descending axon cells (DAC)***. DAC neurons were easily distinguished due to the presence of one to a few descending axon collaterals that reaches layer 4, and 5, occasionally even layer 6 (Figure 1, Table 1). Hestrin and Armstrong have reported similar cells previously in L1. (Hestrin and Armstrong, 1996). Their other striking distinguishing feature is the large horizontal (H: 1035 ± 63 μm) and vertical (V: 602 ± 49 μm) extent of their axonal arborization, which was the largest as compared to any cell type in L1.

***Large axon cells (LAC)***. LAC neurons appear visually similar to the HAC neurons. On closer inspection, however, the axonal segments were shorter and projected more radially compared with HACs. Particularly many short branches emerged from long axonal collaterals. They displayed the longest total length of axon (sum of all axon lengths), the highest number of segments, and the highest maximum branch order of any cell type studied in L1 (Figure 1, Table 1). The axonal collaterals of LACs were often seen to project vertically into layers 2 and 3 (6/10 cells). Their dendrites also displayed the highest segment number (i.e., higher frequency of branching).

***Small axon cells (SAC)***. SAC neurons had the smallest axonal arborization reflected in the lowest total axonal lengths of any cell type (Figure 1). The axonal arbor also displayed the lowest number of axonal segments and the lowest maximum axonal branch order (MABO) (Table 1). The axons had some of the largest axonal branch angles (i.e., more right-angled branching) with the low tortuosity values (i.e., the straightest axonal segments). Few SACs (4/11 cells) projected one or two axonal collaterals into layers 2 and 3.

***Rare cell types***. We found one Cajal-Retzius cell like Cell (CR-like C) consistent with the fact that the CR cell type develops in L1 of very young animals and disappears around P10 (Hestrin and Armstrong, 1996; Portera-Cailliau et al., 2005).

***Relative distribution of L1 cell types***. Based on this subjective classification supported by the statistical significance of distinguishing morphometric features, we report the proportion of cell types present in P14 rat SSC L1:

**Table:** 

**Cell type**	**Total number**	**Percentage (%)**
NGC-DA	33	21.01
NGC-SA	26	16.56
HAC	37	23.56
DAC	25	15.92
LAC	19	12.10
SAC	17	10.82

#### Objective analyses

Recent subjective classification methods for neuron morphologies have been refined and supported by identifying significant morphometric features, as we have performed. Thus far it has only been possible to use unsupervised clustering methods to objectively separate the obviously different pyramidal and non-pyramidal neurons (Guerra et al., 2011). Subtle differences between very similar groups of cells are difficult to distinguish with a solely objective classification and thus, despite major advances, automatic classification of neuronal morphologies is still an unsolved problem. We used PCA and LDA on the morphometric features to identify combinations of key distinguishing features that can be used to objectively classify L1 interneuron morphologies.

**Figure 2:**
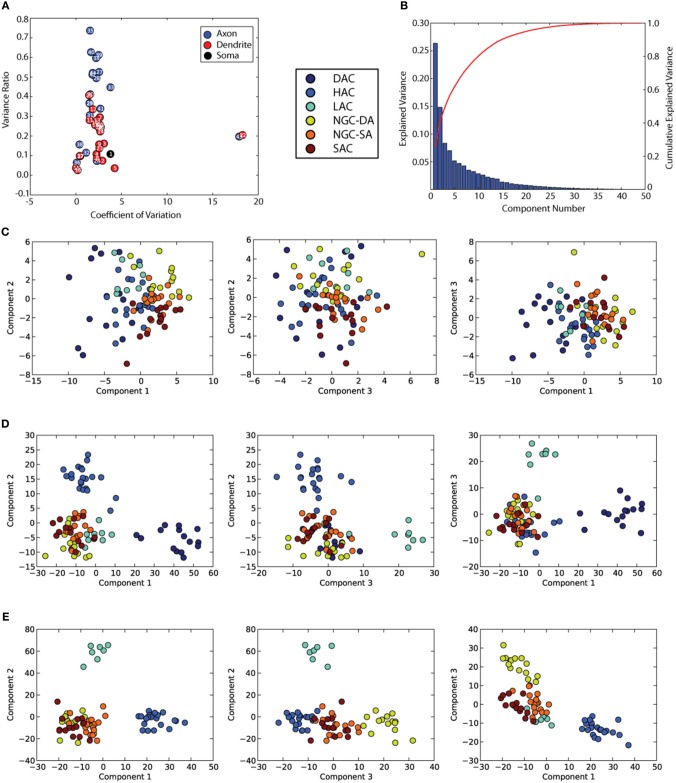
**Objective Morphological analyses of cells found in L1. (A)** shows the feature power analyses which rates mostly axonal features as the most discriminative for classifying L1 cells. **(B)** Shows the amount of variance explained by the PCA components. **(C)** is the set of PCA plots done on the standardized dataset **(D)** is the set of LDA plots (with number of groups = 6) which shows clear grouping of DAC, HAC, and LAC. **(E)** Shows the set of LDA plots with number of groups = 5 (after removal of DAC) and a better separation of NGC-DA.

***A set of morphometric features***. An extensive morphometric analysis was performed on the neuron morphologies resulting in a total of 44 features including those that quantify axonal, dendritic and somatic properties (Table 2). Most of the features listed have been measured in previous analyses of neuron morphologies (Wang et al., 2002). We excluded 2 features that displayed high co-efficient of variations on the feature power plot (Figure 2A, features 43 and 22) (See Table 2). Of the 91 cells studied, the value for all 42 features lay within 2 standard deviations of the mean except for two cells. The dendrite density values (feature 21) for these two cells were treated as outliers due to high values of standard deviations from the mean (8.93 and 2.57). These two cells were excluded from our subsequent analyses.

***Principal component analysis (PCA)***. We first performed a PCA as an objective and unsupervised clustering method. The raw values of the feature dataset were *z*-normalized (around the mean) before applying the PCA. We found that PCA could not generate any meaningful clusters (Figure 2C) and that it could only account for about 25% of the total variance in the first principal component (Figure 2B). This suggests that objective unsupervised classification of neuronal morphologies is currently not possible, based on any single independent feature of neuronal morphology in L1. These results also suggest that other similar unsupervised methods are unlikely to perform better, only marginally better if at all.

***Linear discriminant analyses (LDA)***. LDA is a supervised method that reduces the dimensionality of a given dataset, while at the same time, preserving as much discriminatory information as possible i.e., maximizing the ratio of between-class to within-class variance. LDA which is similar to PCA, but specifically, tests the differences between the hypothesized classes. The result is a weight vector which, when applied to features, produces the maximum possible separation between groups (Krzanowski, 2000).

We provided the hypothesized six classes identified in the subjective, morphometric-supported classification as the target in the LDA. The HAC, DAC, and LAC cells separated very clearly (Figure 2D). A 10 fold validation produced the same clusters. We then removed two of the most polarizing clusters (DAC and HAC) and repeated the LDA with a hypothesis of 4 clusters. We found that the four clusters separate clearly with a high level of cross-validation (see Methods and Supplementary Figure 6). (Figure 2E).

### Electrophysiological classes in layer 1

Compared to cortical Pyramidal Cells, L1 cells are harder to maintain in an electrophysiologically viable state for long periods of time—partly due to their small size (Zhou and Hablitz, 1996). Small cells are also more vulnerable to physical damage (shrinkage and/or swelling) and loss of gigaohm seals. For our study, we were nonetheless able to select 98 cells out of 810 with AP amplitude greater than 50 mV and access resistances less than 10 MΩ.

#### Classical accommodating cells (cAC)

Accommodating Cells that begin to spike with the onset of stimulus, which do not produce bursts and which do not display a delayed response, were classified as cAC (Supplementary Figure 1). 10% of the L1 cells in our sample (*n* = 11) belonged to this firing type.

#### Classical non-accommodating cells (cNAC)

Non-Accommodating Cells, which begin to spike with the onset of stimulus, which do not produce bursts and which do not display a delayed response, were classified as cNAC (Supplementary Figure 2). These were the most common firing-type among L1 cells (*n* = 41, 40%).

#### Bursting non-accommodating cells (bNAC)

Non-accommodating cells that display a burst on the onset of stimulus are defined as bNAC (Supplementary Figure 3; bNAC; *n* = 25). We identified two subtypes of these cells. The first subtype (*n* = 15) consists of bNACs that respond to the stimulus with four rapid spikes with a mean ISI of less than 25 ms.

A second subtype of bNAC (*n* = 10), displayed a “doublet” of spikes (a pair of APs with a maximum ISI of 25 ms) at the beginning of firing responses.

#### Classical stuttering cells (cSTUT)

Cells with a poorly fitted regression line (sum of RMS error greater than 30; mean = 339.05) and at least one “silent period” of more than 100 ms were classified as cSTUT. 13% of L1 cells (*n* = 14) belonged to this class. The average duration of the silent periods was 193.43 ms and the average ISI during spiking was 38.2 ms (Supplementary Figure 4).

#### Classical irregular spiking cells (cIR)

Cells with a poorly fitted regression line (mean of the RMS error = 46.46), which were not classified as cSTUT, were classified as cIR. 7% of L1 cells (*n* = 8) belonged to this class (Supplementary Figure 5).

It should be noted that our classification does not reproduce all the firing-types identified by other authors, for instance the Late Spiking (LS) cells identified by Chu et al. (Chu and Hablitz, 2003). We observe that several alternative classifications are based on the use of stimulation currents around the threshold value. In our own work, we observed that repeated stimulation with these currents produces highly variable spiking behavior. We therefore used a square pulse stimulus of 50 pA and scaled the amplitude of the pulse until it produced a single AP. This procedure ensured replicability, producing a reliable and parsimonious classification scheme. The electrophysiological parameters extracted from the single cell dataset are listed in Table 4.

**Table 4:** **Table of the *p*-values (Students' *t*-test) of comparisons between electrophysiological groups in Table 3**.

**E-code parameters**	**cNAC vs. bNAC**	**cAC vs. bNAC**	**bNAC vs. IR**
AP0 amplitude	0.50	0.45	0.09
AP0 duration	0.14	0.85	0.33
AP0 half-width	0.36	0.95	0.28
AP0 rise-time	0.94	1.00	0.70
AP0 fall-time	0.06	0.82	0.25
AP0 ahp-time	0.02^*^	0.17	0.87
AP0 rise-rate	0.80	0.56	0.09
AP0 fall-rate	0.23	0.20	0.02^*^
AP0 fast AHP	0.01^*^	0.01^*^	0.16
AP1 amplitude	0.85	0.14	0.31
AP1 duration	0.03^*^	0.47	0.25
AP1 half-width	0.03^*^	0.52	0.33
AP1 rise-time	0.44	0.77	0.72
AP1 fall-time	0.01^*^	0.43	0.19
AP1 ahp-time	0.06	0.32	0.85
AP1 rise-rate	0.43	0.24	0.20
AP1 fall-rate	0.02^*^	0.07	0.03^*^
AP1 fast AHP	0.15	0.02^*^	0.45
Input resistance for peak	0.13	0.97	0.05
Input resistance for steady-state	0.13	0.57	0.07
Time constant for delta pulse	0.00^**^	0.03	0.10
AP threshold	0.40	0.97	0.34
sAHP maximum	0.17	0.05	0.20
Minimum current to threshold	0.80	0.61	0.77

*Shown are the group comparisons with maximum parameters with significant p-values*.

* p < 0.05;

** *p < 0.01*.

### Morpho-electrical types

**Figure 3:**
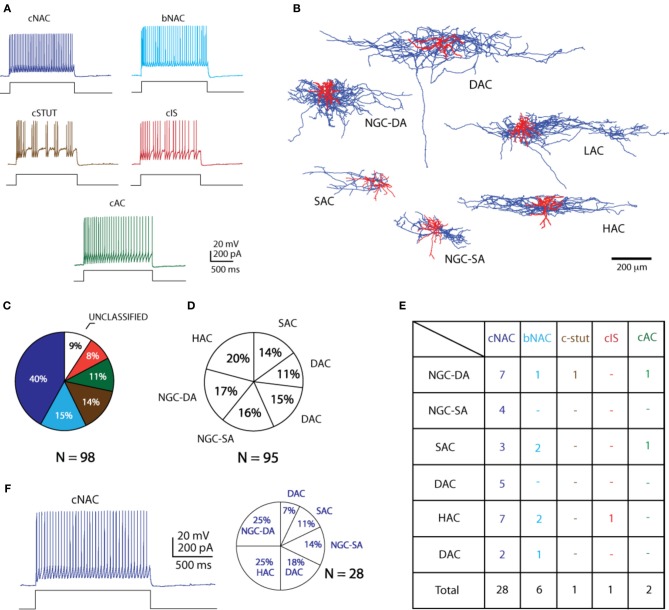
**Morpho—electrical types in L1. (A)** Summary of all the firing patterns and **(B)** all the morphological types found in L1 **(C)** shows the percentage distributions of the electrophysiological subtypes whereas **(D)** shows the morphological subtypes. Firing types pie chart color coded as per types in **(A)**. **(E)** Table showing the distribution of morpho-electrical types found in L1. **(F)** An example case of the most common firing type, cNAC, with the distribution of morphologies.

The combination of the morphological and electrophysiological classification provides a more comprehensive view of the diversity of cells. Of the total number of cells, with both high quality staining and electrophysiological recordings (*n* = 38), 28 (73.6%) expressed a cNAC firing pattern. HAC and NGC-DA were the most commonly found morphological types (10/38; 26.3% each). Combining these two parameters, the most common morpho-electrical type (ME-type) in L1 is observed to be the NGC-DA—cNAC along with the HAC-cNAC combination (7/38; 18.4% each) followed by LAC and NGC-SA under the same firing type (Figure 3).

### Electrophysiological circuitry

Multi-neuron patch-clamp recording from pairs of neurons detected 82 gap junctions and 248 synaptic connections, all displaying hyperpolarizing GABAergic-like IPSPs. For the study reported below, we selected cells with AP amplitudes greater than 50 mV and Access Resistance less than 10 MΩ.

#### Electrical connections

**Figure 4:**
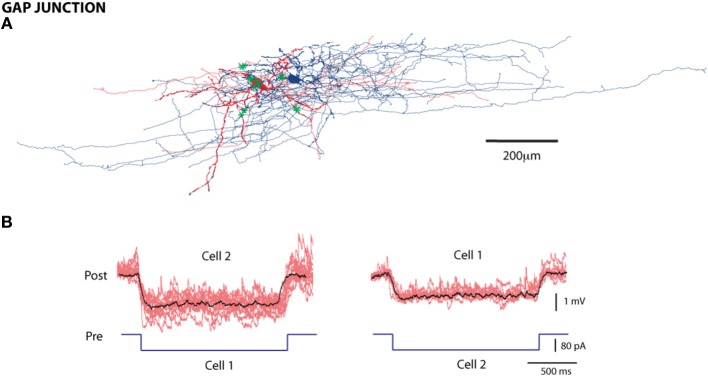
**Gap Junctions between L1 cells. (A)** Reconstruction of a pair of cells connected by gap junctions; green asterisks are the contact points. Scale bar = 200 μm **(B)** An example of a gap junction with electrical traces; individual traces in red and the average in black, with the hyperpolarizing stimulus reflected in both cell 1 and 2.

To detect gap junctions, we injected a hyperpolarizing step current pulse (50–100 pA) into one cell in each pair and measured the hyperpolarized response from the other (Figure 4A). The average coupling co-efficient (the ratio of the amplitude of the post-synaptic response to the pre-synaptic response) was 0.0543 ± 0.028 (mean ± *SE*). All recorded gap junctions (82/1568 pairs; 5.2% probability); conducted bi-directionally, with no significant differences between conductances in the two directions (coupling coefficients 0.052 ± 0.02 and 0.056 ± 0.03 respectively) and were therefore classified as symmetrical gap junctions.

#### Synaptic connections

For further analysis, we selected 142 out of 248 connections with IPSPs >1 mV after 5 repetitions of the stimulation protocol.

**Figure 5:**
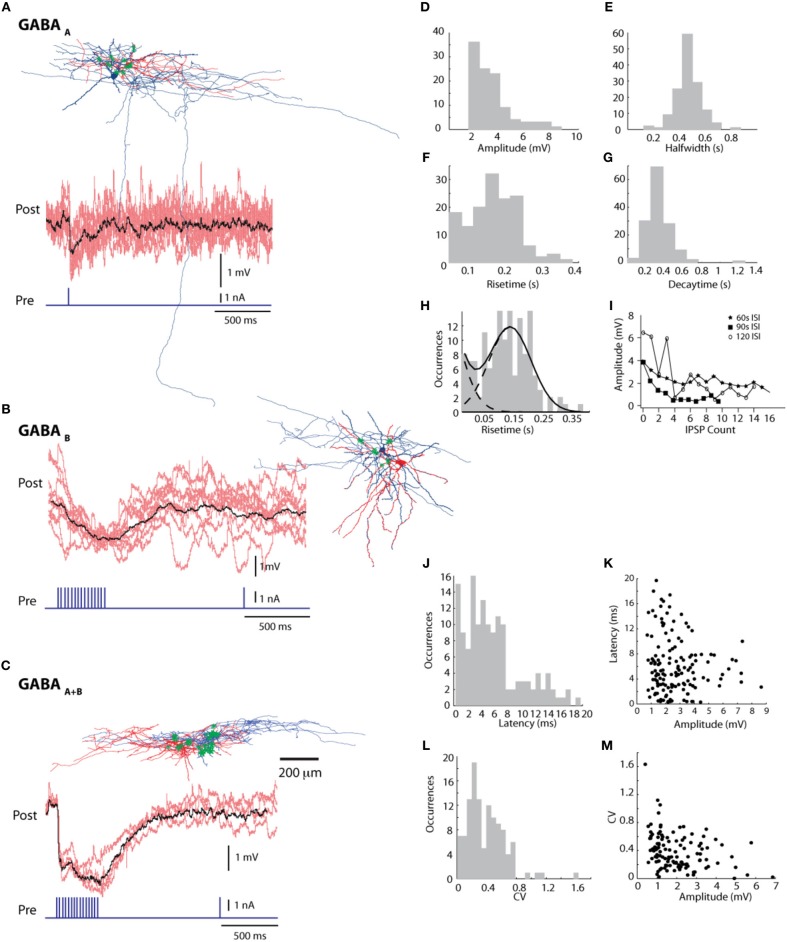
**Synaptic connections between L1 cells. (A)** GABA_A_, **(B)** GABA_B_, and **(C)** GABA_A+B_ connections; individual traces in red and mean in black. Reconstruction of a pair of cells with for each type connection— pre-synaptic in blue and post-synaptic in red, points of contact marked with green asterisks. See Supplementary Figure 7 for positions of putative contact points. Histograms of synaptic parameters—**(D)** amplitude, **(E)** halfwidth, **(F)** risetime and **(G)** decaytime. **(H)** The risetime histogram fitted with a double Gaussian histogram, indicating the presence of two possible populations of GABAergic connections **(I)** decay of IPSP amplitude over stimulation repetitions with 3 different Inter stimulus Intervals (ISI) **(J)** distribution of latency values **(K)** Latency vs. amplitude graph with a linear fit and a slope of −0.32 **(L)** Distribution of values of Co-efficient of Variation (CV) **(M)** CV vs. IPSP amplitude graph showing a decrease in CV value with increase in amplitude.

In 31 connections a single AP was sufficient to evoke IPSPs, always with rise times less than 60 ms (28.7 ± 15 ms; mean ± *SE*) (Figure 5A). However, the majority of connections (*n* = 110) only responded to trains of stimuli, at a minimum frequency of 40Hz, usually but not always with rise times between 60 and 379 ms (159.458 ± 60.239 ms; mean ± *SE*).

An analysis of IPSP latencies (Figures 5D–M) shows a distinction between a group of connections with a mean latency of 4.148 ± 2.479 ms (mean ± *SE*, *n* = 111), and the other with a mean of 12.89 ± 2.963 ms (mean ± *SE*, *n* = 32) (Figure 5J). Decay times varied between 105 ms and 1.26 s (336.2 ± 138.7 ms; mean ± *SE*). The CV (coefficient of variation) had a mean value of 0.386 ± 0.252 (mean ± *SE*).

Fast rise times and low latencies are characteristic of GABA_A_ transmission dynamics. Contrarily, slow rise times and higher latencies suggest a role for GABA_B_ transmission, a hypothesis supported by our calculated potassium reversal potential (−102 mV). However, we also observed that in many cases connections with fast rise times and low latencies had reversal potentials close to the typical values for potassium and that connections with slow rise times and high latencies often had reversal potentials around the typical values for chloride.

**Figure 6:**
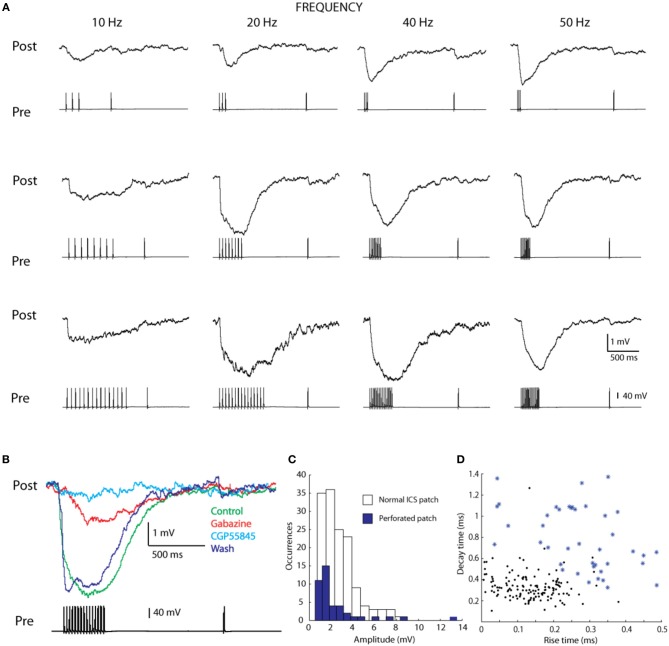
**Perforated patch of L1 cells. (A)** A frequency analyses of the synaptic connection performed with perforated patch, examples showing variation in spike number (3, 8, and 15) and frequency (10, 20, 40, 50 Hz) **(B)** pharmacology performed with Gabazine (GABA_A_ blocker) and CGP55845 (GABA_B_ blocker). **(C)** Histogram of the amplitudes of connection performed with normal ICS patch and perforated patch, showing the detection of lower amplitude connections with perforated patch. **(D)** Rise and decay times of the connection also differ, possibly due to the perforated patch.

To clarify the respective contributions of GABA_A_ and GABA_B_ receptors we performed 44 experiments with perforated patch clamping, a technique that limits cytoplasmic dilution and eliminates amplitude decay. In the seven pairs of connected cells, for which it was possible to obtain stable recordings (pre drug IPSP amplitude = 4.83 mV), we subjected the connection to a set of pre-synaptic train stimuli of varying frequencies (Figure 6A). We also applied specific inhibitors, and measured the resulting IPSPs (Figure 6B). The presence of a GABA_A_ antagonist (20 μ M Gabazine) led not only to reductions in IPSP amplitudes but also completely abolished fast rise times (IPSP amplitude during Gabazine perfusion = 1.66 mV). The introduction of a GABA_B_ antagonist (4 μ M CGP55845) blocked the remaining IPSP amplitude (IPSP amplitude during CGP55845 perfusion = 0.64 mV). After washout, the amplitude of the IPSP recovered almost completely (IPSP amplitude after washout = 3.27 mV) (Figure 6B).

These results prove that the GABA_A_ and GABA_B_ receptors are both involved in mediating the synaptic response. The GABA_A_ receptors are responsible for fast rise times and most of the amplitude of the IPSP and the GABA_B_ receptors are responsible for slow rise times.

### Morphological circuitry

#### Gap junctions

To verify the morphological cell types involved in gap junctions, we selected four electrically coupled and stained cell pairs. Of the four pairs, one involved LACs; two pairs involved a LAC and a HAC, and a LAC, and a DAC, respectively; one involved a DAC and an NGC-DA. We reconstructed one electrically and synaptically coupled L1 pair (Figure 4). Among the eleven putative coupling contacts, four were on the dendrites. Seven contacts involved the axonal collaterals of the two neurons.

#### Synaptic connections

Among eighteen pairs of connected L1 neurons from fourteen well-stained arbourisations, four neuron types (HAC, DAC, NGC-DA, and LAC) were found to form GABAergic synaptic connections. In most cases, synapses were between neurons of the same type (11/18 pairs, 61%), mainly HACs and/or DACs (8/11 pairs, 73%), or NGC-DAs (3/11 pairs, 14%). All connections between different types of neurons (7/18 pairs, 39%) involved HAC and/or DAC cells, mostly as post-synaptic cells (5/7 pairs). LAC (3/7 pairs) and NGC-DA (2/7 pairs) were often pre-synaptic cells.

Five pairs of L1 connected neurons were reconstructed. These cells had an average of 9.2 putative synapses per pair on the soma and dendrites of the post-synaptic cell. A high fraction (39%) of putative synapses was formed on the soma. This kind of contact was observed in four out of five reconstructed pairs. (Figures 5A–C for the cell pairs and Supplementary Figure 7 for zoomed in views).

## Discussion

Literature shows that there have been very few studies on the constituent cells and connections of neocortical L1. Most of the results have produced unique methods of classification, ultimately resulting in a discordant scheme. With a view of integrating L1 with the rest of the laminae in the cortical column, it is not possible to clearly understand the contribution of L1 to cortical activity, with the current data available.

In this study, we characterize the single cell and microcircuit properties of the somatosensory cortical L1 of juvenile rat, using standardized protocols for single and multi-electrode patch clamp electrophysiology, biocytin labeling and immunohistochemistry and introduce reproducible methods for the classification of electrophysiological behavior and neuron morphologies. We identified six morphological classes of neurons, all present with approximately equal frequencies. More than 60% of these cells displayed a Non-Accommodating (NAC) pattern of firing. As already seen in previous studies, some connections among these neurons were recruited by single APs, displaying IPSP kinetics reminiscent of GABA_A_ mediated responses. However, a majority was not activated by single APs and required high frequency trains of pre-synaptic APs. This is a novel finding. In some cases, the observed IPSPs were similar to GABA_B_ mediated responses. In others they showed rise time properties characteristic of GABA_A_ in combination with slow-decay times characteristic of GABA_B_. Subsequent pharmacological manipulations confirmed that both GABA_A_ and GABA_B_ receptors were implicated in the IPSP kinetics.

### Development of immunohistochemical marker expression

A very recent detailed study by Martinez-Galan et al. showed the expression patterns of calcium binding proteins in L1 over developmental time points. Comparing the developing rat and mouse neocortex, they found clear differences in the patterns of marker expressions. In contrast with the developing mouse, the rat L1 at P14 was observed to contain a majority of cells positive for Reelin but very few cells that are also positive for Calbindin or Calretinin (Martinez-Galan et al., 2013).

Along with calcium binding proteins, L1 neurons also seem to be mostly lacking in Parvalbumin and the interneuronal peptides such as Somatostatin (SOM). On the contrary, Karagiannis et al. have shown that neurons in juvenile L1 express NPY. Along with establishing a strong base for the classification of the NPY positive neurons in the cortex, their data subdivides this neuronal population into three major subtypes—FS parvalbumin positive interneurons, Martinotti-like somatostatin positive interneurons and finally, Ngf cells (Karagiannis et al., 2009). Combining this information and data from our studies on L1, we can conjecture that the Ngf cells that we have classified should be positive for NPY.

### Single cell properties

#### Morphology

In our classification of L1 morphologies we developed an initial subjective classification, and subsequently refined it using objective methods. This approach allowed us to identify six distinct morphological groups, a finer classification scheme than those developed by other authors, who reported between 2 and 4 groups.

Our classification final scheme includes three morphological types (NGCs, HACs, and DACs) previously identified by Hestrin and Armstrong (1996). Unlike these authors, however, we split the NGCs into two (NGC-SA, NGC-DA) groups and add two completely new cell types (SAC and LAC).

Like our electrophysiological classification, the methods we have used in this study are fully standardized and lend themselves to work in other layer and brain areas.

We recognize that some aspects of our current classifier are stronger than others. In particular, there is a very clear distinction between HAC, DAC and the rest of the other neurons. However, the differences that distinguish NGC-SA and NGC-DA are relatively subtle and the functional relevance is an open question.

#### Electrophysiology

In our study, as in previous work by Zhou and Hablitz, small L1 interneurons were frequently damaged or destroyed due to post-recording pipette withdrawal (Zhou and Hablitz, 1996), leading to physical damage (shrinkage and/or swelling) and loss of gigaohm seals. Despite the loss of a significant number of cells, we were nonetheless able to select a final single cell dataset of 98 cells.

Our analysis revealed that all these cells could be placed into classes based on the firing patterns described by the Petilla interneuron convention (Ascoli et al., 2008). Like the previous study (Hestrin and Armstrong, 1996), we detected non-accommodating cNAC cells (40% of the cells in our study). Unlike these studies, we also found several other types of cell including bNAC (15%), cSTUT (14%) cAC (11%), cIR, (8%) and unclassified firing types (9%). The study did not detect the FS cells reported in (Wozny and Williams, 2011). One of the reasons may be the use of rats older than P13–P16. Higher currents would normally be expected to induce higher firing frequencies. However the physiological relevance of observation made under these conditions is questionable.

Similar considerations apply to the Late and Non Late spiking cells, reported by Chu et al. (2003). As reported earlier, our work used a standardized current injection protocol, in which currents were scaled to produce a regulated firing frequency. This means we do not have to rely on the firing threshold, which is known to be highly variable. Chu et al, by contrast, do not attempt to maintain such a standard. It is possible, therefore, that the different spiking behaviors they observe could be the result of uncontrolled changes in threshold values. During our own experiments, we sometimes observed apparently random changes from late to non-late spiking behavior and vice versa (data not shown). This supports the idea that early and late spiking behavior is not sufficiently stable to be used as a criterion for electrophysiological classification of neuron types.

We believe that the methods we have applied in L1 are suitable for use with other cell populations, for example in other layers and/or areas of the cortex and in other species. In particular, we believe it is especially important to focus on firing patterns since it makes it possible to compare data for a specific population of interneurons with data for other populations as classified by the Petilla Convention (Ascoli et al., 2008).

Although the distinctions between different firing patterns were relatively clear, we are aware that our current classification is not definitive. However, these distinctions, on their own are not enough to detect the way individual ion channels affect the electrophysiological behavior of the cell. This is a theme for future research that will probably require the development of new protocols.

### Circuit properties

To our knowledge, there has only been one previous study (Chu et al., 2003) of the circuit properties of these L1 interneurons.

One of our main findings is the discovery of slow transient GABA_A+B_ mediated IPSPs between neocortical L1 cells. This matches similar phenomena previously observed in the ferret thalamocortical loop (Kim et al., 1997), in rat neocortical layer 5 and the CA1 region of hippocampus (Thomson and Destexhe, 1999) and in connections between cortical interneurons and pyramidal cells in other layers (Chu and Hablitz, 2003; Tamás et al., 2003; Pérez-Garci et al., 2006; Suzuki and Bekkers, 2010a,b).

A majority of these slow inhibitory connections required a 40 Hz spike train to produce a post-synaptic response. To our knowledge, no other study has documented this kind of post-synaptic GABA_B_ response between L1 cells in the neocortex. Rise times varied between 5 ms and 379 ms. Response amplitudes decayed rapidly with increasing number of repetitions. We hypothesized that the decrease was due to the dilution of intracellular contents with the ICS, leading to the depletion of secondary messenger molecules such as cAMP (cyclic adenosine monophosphate). This interpretation is supported by the rapid decline in the amplitude of the IPSPs observed during repeated stimulation—exactly what we would expect with depletion of secondary messenger molecules. Perforated patch clamp experiments—which conserve the intra-cellular environment - showed no comparable decay. This is confirmatory evidence for our hypothesis.

These observations can be explained in terms of a dynamic molecular model of the binding of GABA to its receptors proposed by Destexhe and Sejnowski (1995). In this model, as in our own observations, IPSP responses have a slow sigmoidal rising phase and a multi-exponential decay. The authors explain this delay by the density of co-releasing terminals and the number and frequency of pre-synaptic APs. The model IPSPs also display a 10–20 ms onset delay, similar to the delay observed in our own experiments. Destexhe and Sejnowski explain this second delay by the time necessary for multiple G protein binding sites to cooperatively bind G proteins. (Destexhe and Sejnowski, 1995).

### Role of L1 in the neocortex

#### Columnar inputs

Axons from L2/3 and L5 PCs in the primary column ascend to innervate supra granular layers, including L1 (Gottlieb and Keller, 1997; Larsen and Callaway, 2006). Examples of functional L2/3 to L1 connections can be seen in inter-laminar studies (Chu et al., 2003; Wozny and Williams, 2011). Slender tufted pyramidal cells in Layer V of the barrel cortex have dense axonal projections to L1, and other supra granular layers (Oberlaender et al., 2011).

Martinotti cells (MCs), a type of interneuronal class, have axonal projections to L1. Since the density of their axonal arbors varies (Wang et al., 2004), MCs in different layers may regulate the signal processing in L1 to different degrees.

#### Non-columnar inputs

Major cortico-cortical fiber afferents to SSC L1 (SI-L1) arrive from “higher order” areas such as primary motor cortex (MI) and the secondary somatosensory cortex (SII) (Cauller, 1995) in the same hemisphere. Palmer et al. (2012) have shown that ipsilateral L1 and supragranular layers are innervated by Layer 5 Pyramidal cells (L5PC) axons from the same brain region in the contralateral hemisphere. Glutamatergic input from the L5PC in the opposite hemisphere activated the ipsilateral L1 interneurons leading to the direct inhibition of ipsilateral L5PC apical dendrites. Post-synaptic GABA_B_ receptors are responsible by mediating a metabotropic inhibition of active dendritic currents.

#### Long range inputs

Major cortico-cortical fiber afferents to SSC L1 arrive from “higher order” areas such as primary MI and the secondary SSC (Cauller, 1995). SSC L1 receives its thalamocortical input primarily from the posterior nucleus, whereas motor neocortical L1 receives from the ventromedial and ventrolateral nuclei of the thalamus (Oda et al., 2004; Rubio-Garrido et al., 2009). This input is seen to innervate both the primary and the neighboring cortical column (Oberlaender et al., 2011).

One of the first studies to look at *in vivo* recording of L1 inteneurons was by Zhu et al. Continuing with the previous classification from Chu et al. the authors performed an *in vivo* blind patch recording of L1 cells coupled with whisker stimulation in the adult rat (Chu et al., 2003; Zhu and Zhu, 2004). They found that the Local Circuit Neurons (LCNs) and the Deep-Layer-Projecting Neurons (DLPNs) in L1 have different receptive fields in terms of size and acuity. They also performed patch clamping of L5PC apical dendritic tufts to compare and contrast their receptive fields with the latter.

Letzkus et al. points out the functional importance of long-range fibers in combination with constituent L1 activity. They saw that L1 firing frequencies respond to behavioral stimuli, such as foot shock, which activate afferent cholinergic fibers from the basal forebrain that innervate the neocortex. This change in L1 activation was also seen to specifically inhibit L2/3 Parvalbumin positive cells. Thus, the authors showed that L1 mediated disinhibition is vital for learning and processing information in neocortical circuitry (Letzkus et al., 2011).

Cruikshank et al. provide us with a crucial result in sub cortical control of L1 circuits in their work on the prefrontal cortex. With a series of optogenetic experiments, the authors proved that thalamocortical connections from the “matrix” neurons to L1 could transmit strong, fast and high fidelity synaptic signals. These projections were seen to drive the LS subtype of the L1 interneurons, which in turn generated feed forward inhibition to the L2/3 pyramidal cells.

#### L1 outputs

Since majority of the axons of L1 neurons are largely confined to L1, their primary targets should be the tuft dendrites of pyramidal cells of other layers. Studying the L1 morphologies, we see that DACs are the only cells that provide an efferent axonal projection from L1. They also facilitate the formation of “disynaptic” inhibitory connections between across PCs and interneurons in both L2/3 and L5. One of the main results of this study is the discovery of hitherto unknown circuitry spanning intra columnar L1 and L2/3 interneurons and L5PCs. Apart from establishing a basic classification scheme for L1 cells, the authors have elucidated an elaborate scheme of inhibition and disinhibition within the said circuit. Thus, along with the variety of L2/3 interneurons involved, this study provides us with information about inter-laminar intra columnar connectivity patterns (Jiang et al., 2013).

## Conclusion

In the study presented here, we set out to identify and characterize the components and connectivity of neocortical L1 in the SSC of juvenile rat. We believe that we have accomplished this goal. Our results include the identification and classification of the morphologies and electrical behavior of L1 neurons, the quantification of the presence of different morphoelectrical types, the characterization of intra-laminar connectivity, and the identification of a new kind inhibitory connection between L1 interneurons mediated by a combination of GABA_A_ and GABA_B_ receptors.

We also aim to make it possible to connect our results with those from other groups and ultimately, make robust data available for the modeling of the rodent brain by the Blue Brain Project.

## Author contributions

Shruti Muralidhar, and Henry Markram conceived and designed the experiments; Shruti Muralidhar performed the electrophysiology experiments and analyzed the data; Yun Wang analyzed the data from the morphological reconstructions; Shruti Muralidhar and Henry Markram drafted the manuscript. All authors discussed the results and critically commented on the manuscript and all authors approved the final version.

### Conflict of interest statement

The authors declare that the research was conducted in the absence of any commercial or financial relationships that could be construed as a potential conflict of interest.

## Supplementary material

Supplementary Figure 1 **(A)** The characteristic continuous adapting firing pattern. **(B)** Two scatter plots showing the raw values with their linear fits and a grand fit (slope = 1.168 mV/ms) of the same. All cells with slopes clearly greater than 1mV/ms were taken as adapting. **(C)** Typical responses of a cAC cell to various current injection stimuli and **(D)** the 3 major groups of cells that show cAC firing pattern.

Supplementary Figure 2 **(A)** The typical continuous non-adapting firing pattern. **(B)** A scatter plot of the ISI values, with linear fits and the graph on the right showing a fit to the fits. Both linear fits show minimum positive values of slope (−0.013 mV/ms) indicating minimum changes in the ISI values over the period of current injection **(C)** The response of a single cell to various current stimulations, as part of the ecode. **(D)** The typical morphologies of the cells that show the cNAC firing type.

Supplementary Figure 3 **(A)** The characteristic burst non-adapting firing pattern **(B)** The graphs show the abrupt jump in ISI values followed by a linear fit on the consecutive values, resulting in a majority of slopes values lesser than 1 mV/ms (0.322 mV/ms). The black line is the line with the average slope. **(C)** A snapshot of the responses of bNAC cells to various current stimuli and **(D)** the typical morphologies than show the bNAC firing pattern.

Supplementary Figure 4 **(A)** The classic stuttering firing pattern with episodes of sudden action potentials followed by protracted periods of silence. **(B)** A scatter plot showing the ISI values of cSTUT cells reaching up to 700 ms **(C)** Typical responses to characteristic current stimuli injections **(D)** NGC-DA, the only morphology found expressing the cSTUT firing pattern.

Supplementary Figure 5 **(A)** Shows the typical irregular firing pattern (cIR) with irregularly spaced action potentials and **(B)** shows the irregularity in ISI values **(C)** is the typical response to a variety of current stimuli and **(D)** the only morphological type that shows the ISI pattern—the HAC.

Supplementary Figure 6 **(A)** shows a variance analyses heatmap done on the standardized raw data. **(B)** shows the feature power analyses after the removal of two outlier features (22 and 43) describing dendritic and axonal tortuosity. **(C)** shows the LDA performed on the dataset after removal of both DAC and HAC. Both the NGC-DA and NGC-SA along with LAC and SAC are seen to cluster separately. **(D)** shows the feature power analyses for the LDA in **(C)**.

Supplementary Figure 7 **(A)** is the close-up of a 3D rendering of the two cells with gap junctions and all the contact points marked with yellow hexagons. **(B)** for cells connected by GABA_A_ **(C)** for cells connected by GABA_B_ and **(D)** for cells with both GABA_A+B_.
