# Supplementary material for: Synaptic and cellular organization of layer 1 of the developing rat somatosensory cortex
Source: Front Neuroanat. 2014 Jan 16;7:52. doi: 10.3389/fnana.2013.00052 (PMC3893566; doi:10.3389/fnana.2013.00052)
Supplement: Supplementary Figure 1 — (A) The characteristic continuous adapting firing pattern. (B) Two scatter plots showing the raw values with their linear fits and a grand fit (slope = 1.168 mV/ms) of the same. All cells with slopes clearly greater than 1mV/ms were taken as adapting. (C) Typical responses of a cAC cell to various current injection stimuli and (D) the 3 major groups of cells that show cAC firing pattern. [file Presentation1.PDF]

cAC

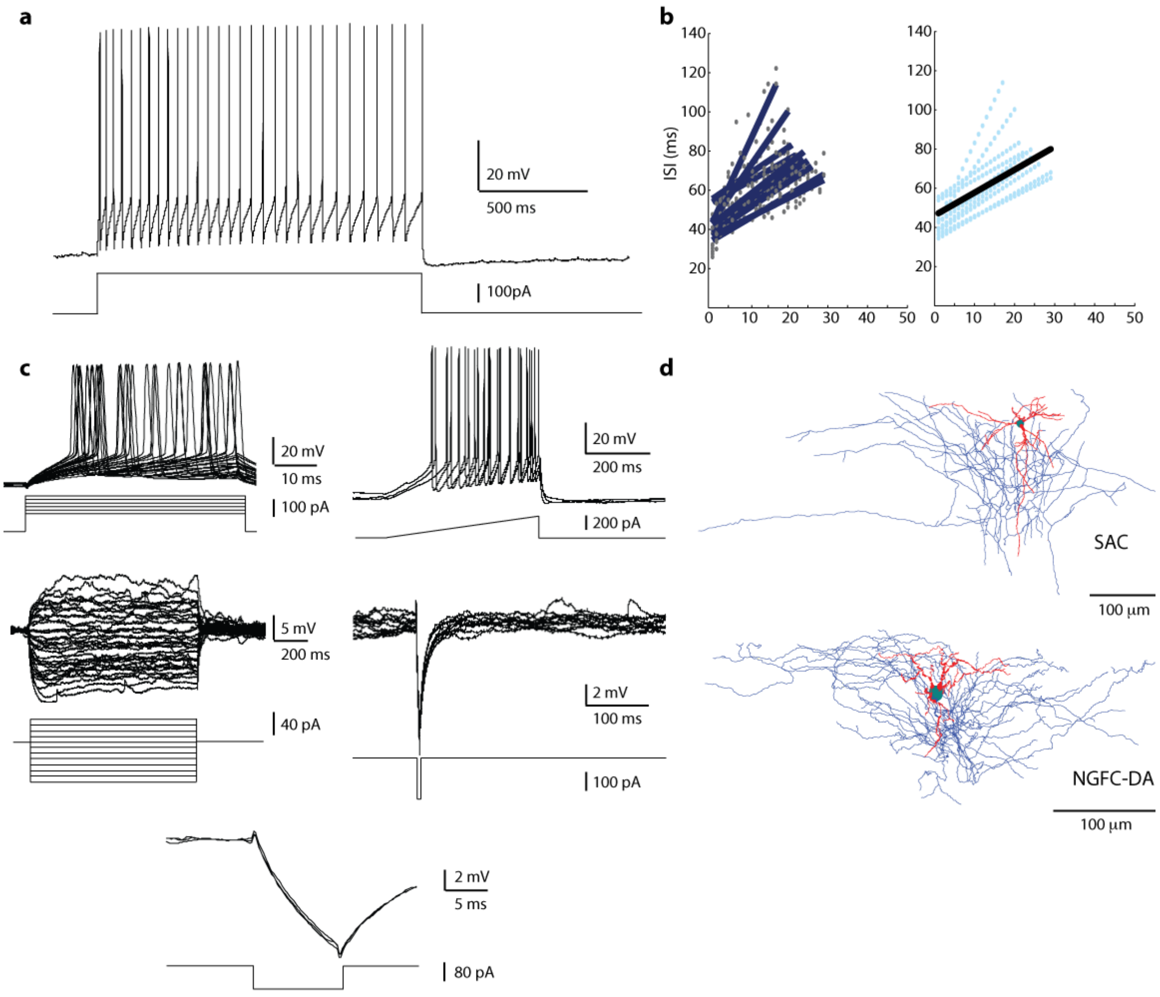

Supplementary Figure 1:

(a) The characteristic continuous adapting firing pattern. (b) Two scatter plots showing the raw values with their linear fits and a grand fit (slope = 1.168 mV/ms) of the same. All cells with slopes clearly greater than 1mV/ms were taken as adapting. (c) Typical responses of a cAC cell to various current injection stimuli and (d) the 3 major groups of cells that show cAC firing pattern.

cNAC

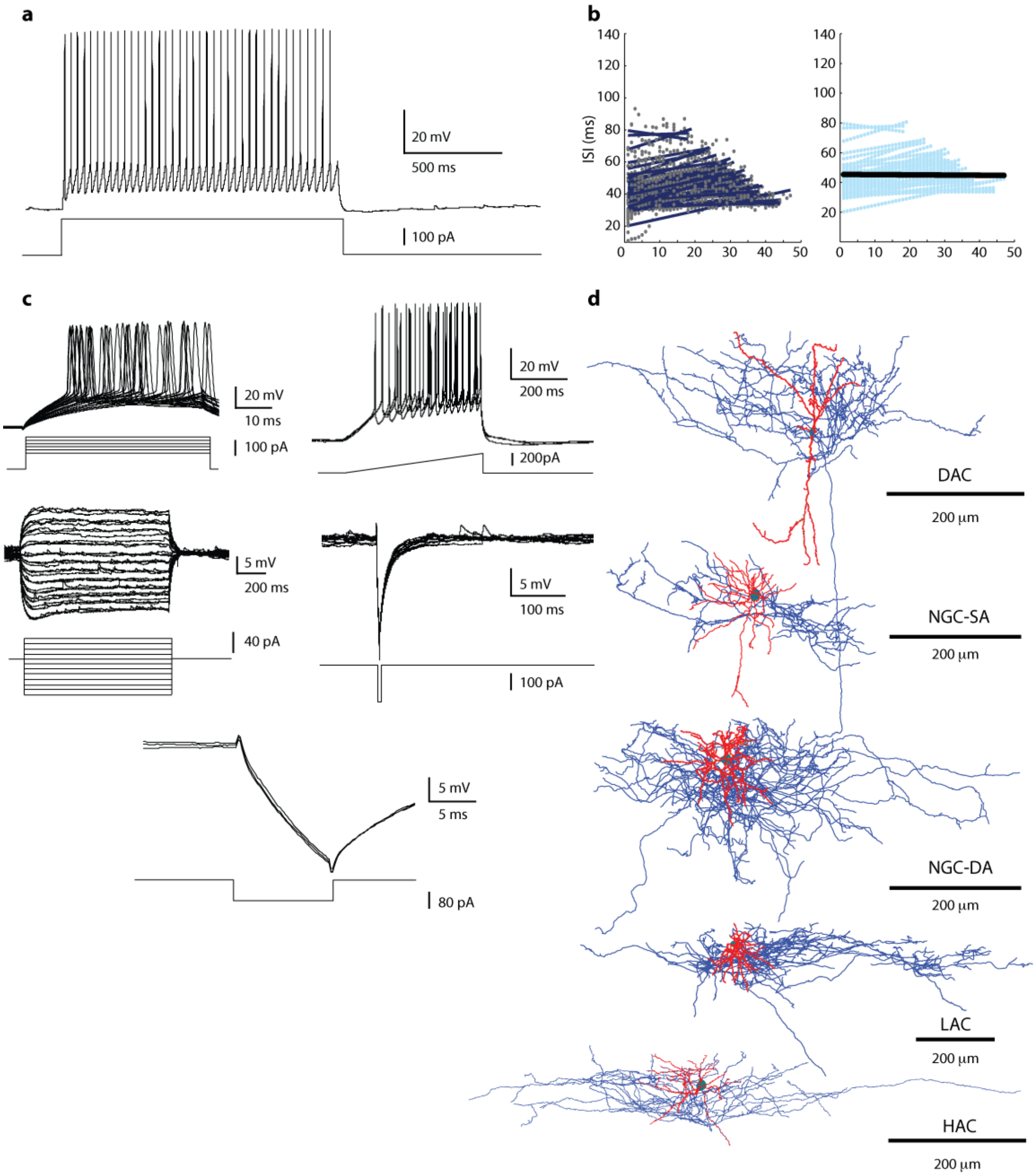

Supplementary Figure 2:

(a) The typical continuous non-adapting firing pattern. (b) A scatter plot of the ISI values, with linear fits and the graph on the right showing a fit to the fits. Both linear fits show minimum positive values of slope (- 0.013 mV/ms) indicating minimum changes in the ISI values over the period of current injection (c) The response of a single cell to various current stimulations, as part of the ecode. (d) The typical morphologies of the cells that show the cNAC firing type.

bNAC

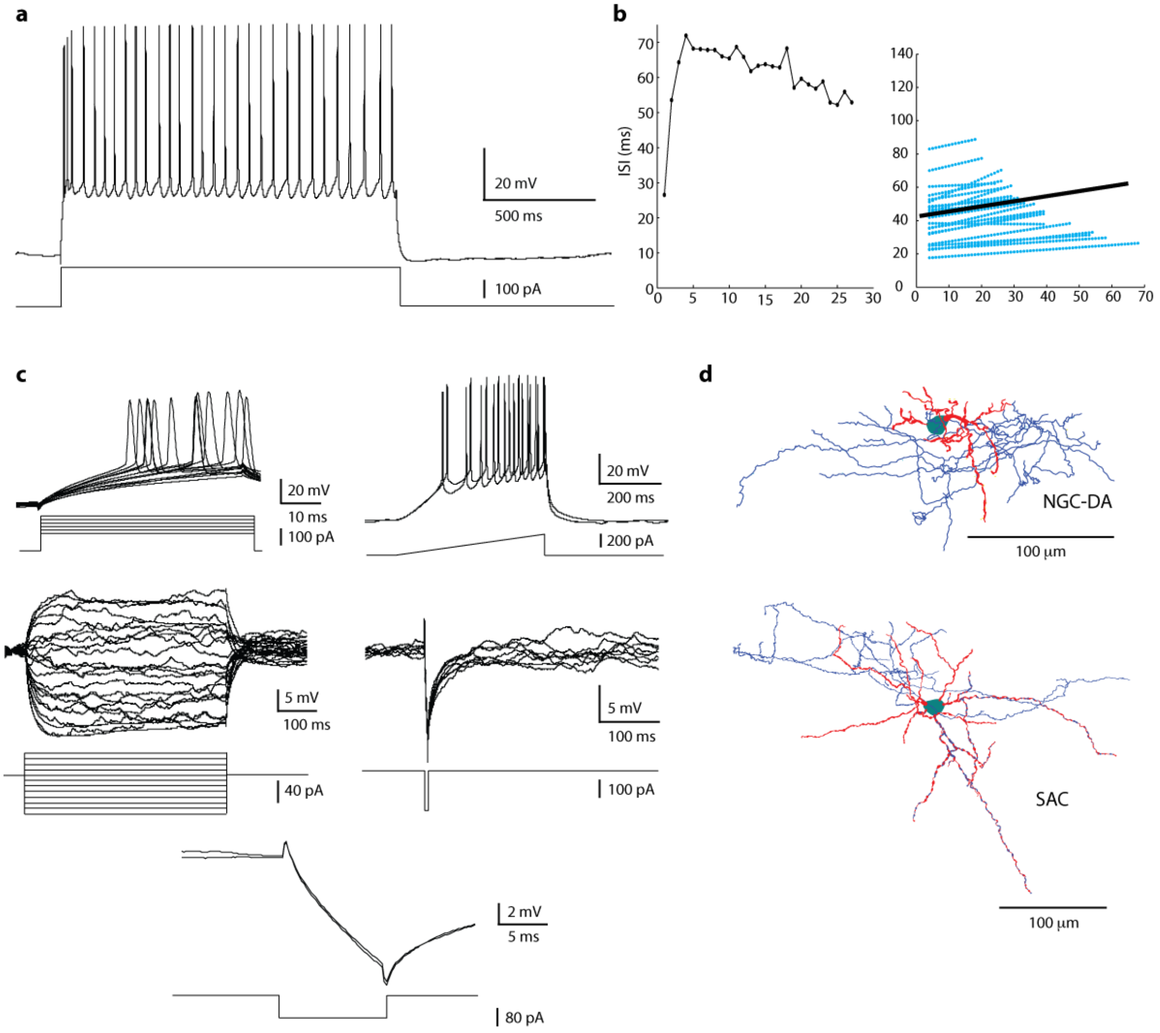

Supplementary Figure 3:

(a)The characteristic burst non-adapting firing pattern (b) The graphs show the abrupt jump in ISI values followed by a linear fit on the consecutive values, resulting in a majority of slopes values lesser than 1 mV/ms (0.322 mV/ms). The black line is the line with the average slope. (c) A snapshot of the responses of bNAC cells to various current stimuli and (d) the typical morphologies than show the bNAC firing pattern.

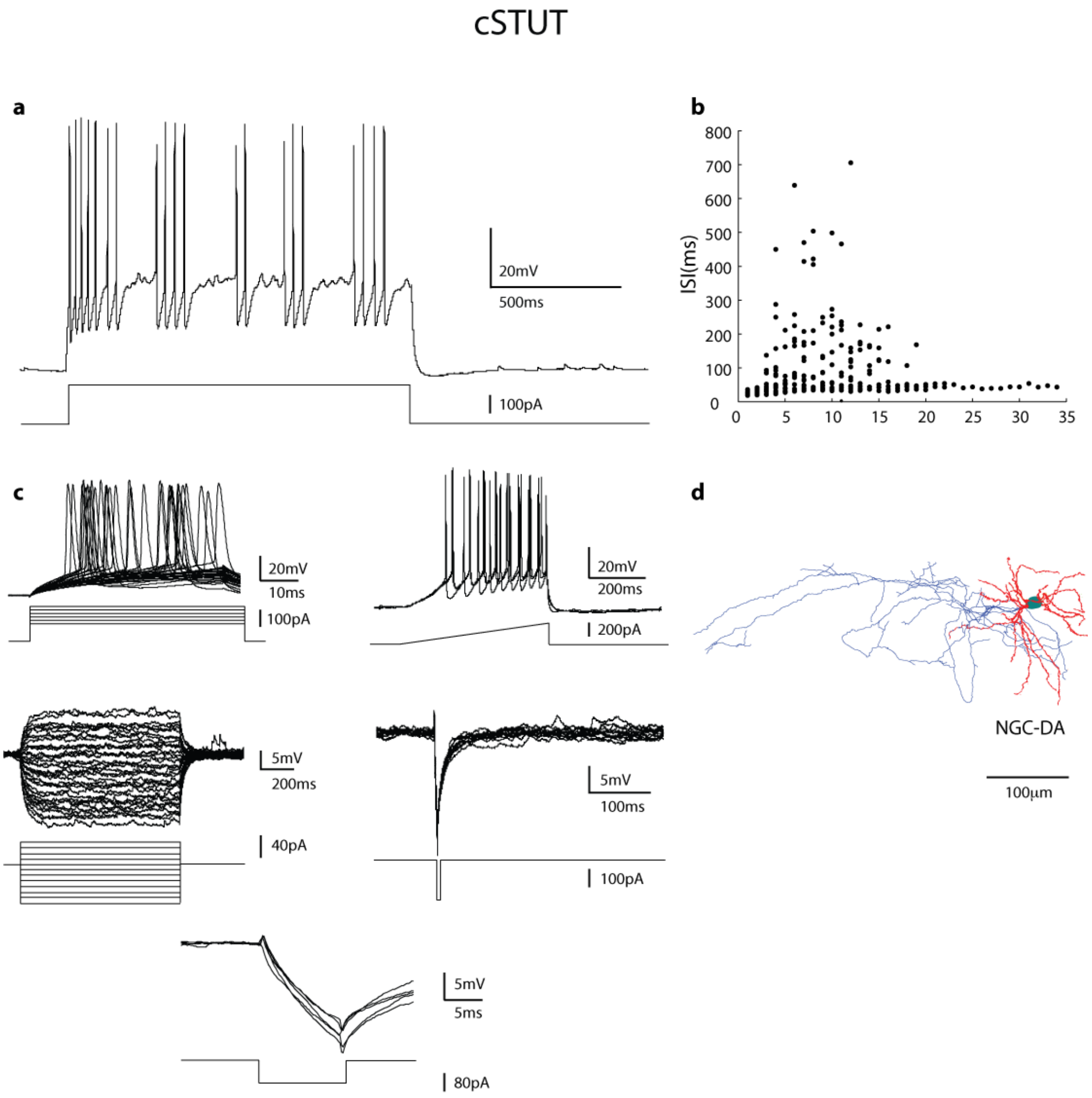

Supplementary Figure 4:

(a) The classic stuttering firing pattern with episodes of sudden action potentials followed by protracted periods of silence. (b) A scatter plot showing the ISI values of cSTUT cells reaching up to 700 ms (c) Typical responses to characteristic current stimuli injections (d) NGC-DA, the only morphology found expressing the cSTUT firing pattern.

cIR

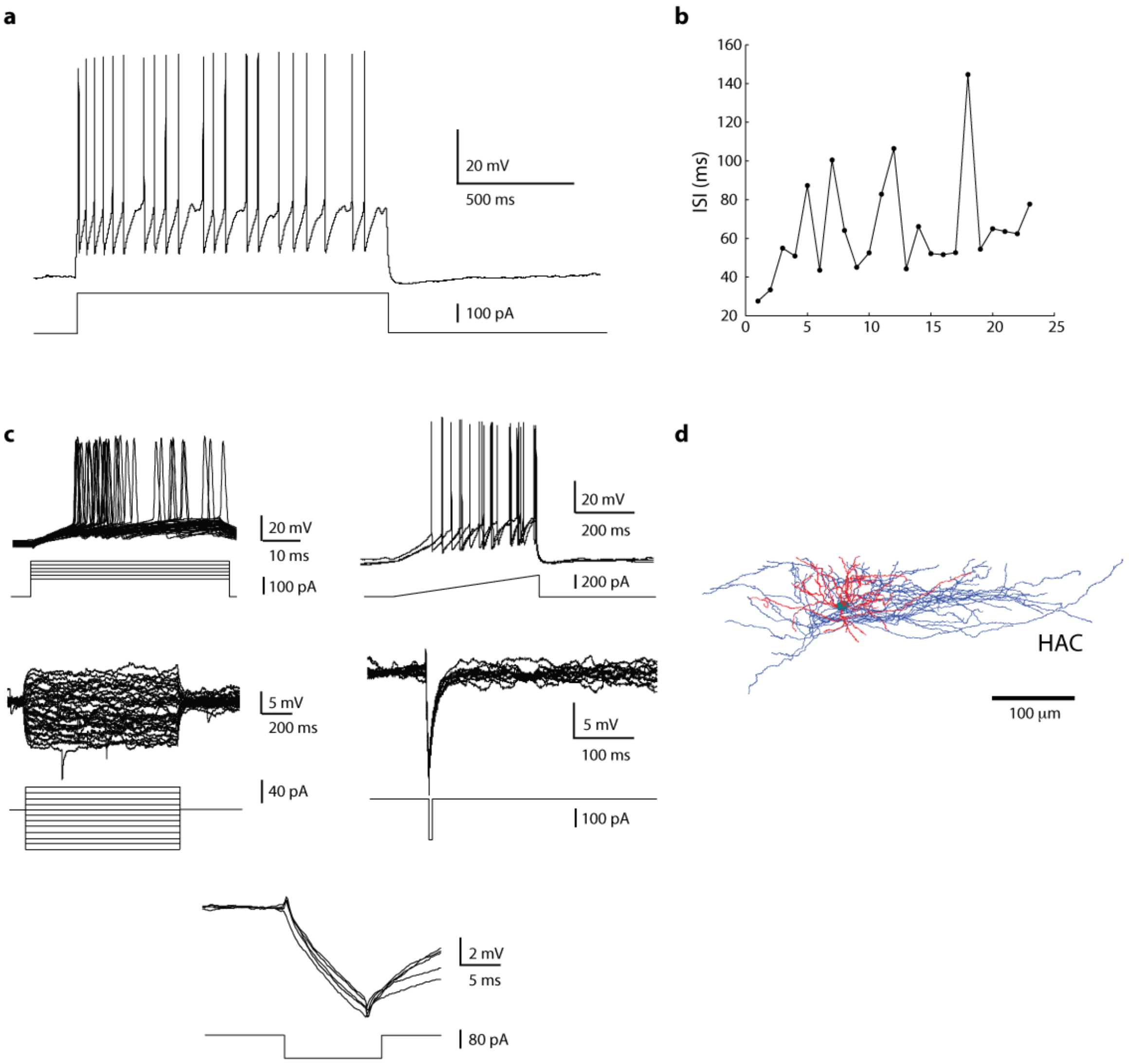

Supplementary Figure 5:

(a) shows the typical irregular firing pattern (cIR) with irregularly spaced action potentials and (b) shows the irregularity in ISI values (c) is the typical response to a variety of current stimuli and (d) the only morphological type that shows the ISI pattern – the HAC.

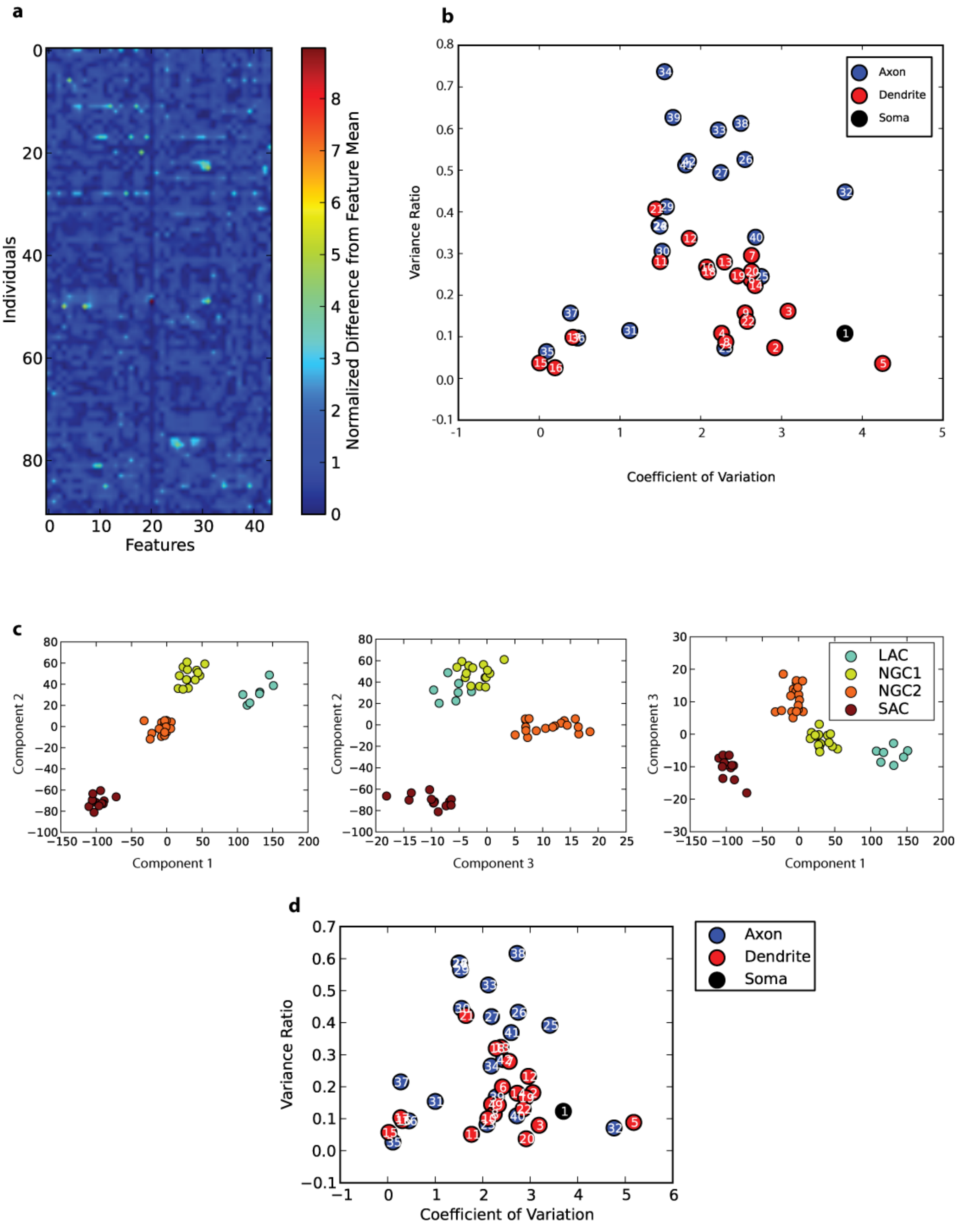

Supplementary Figure 6:

(a) shows a variance analyses heatmap done on the standardised raw data. (b) shows the feature power analyses after the removal of two outlier features (22 and 43) describing dendritic and axonal tortuosity. (c) shows the LDA performed on the dataset after removal of both DAC and HAC. Both the NGC-DA and NGC-SA along with LAC and SAC are seen to cluster separately. (d) shows the feature power analyses for the LDA in (c).

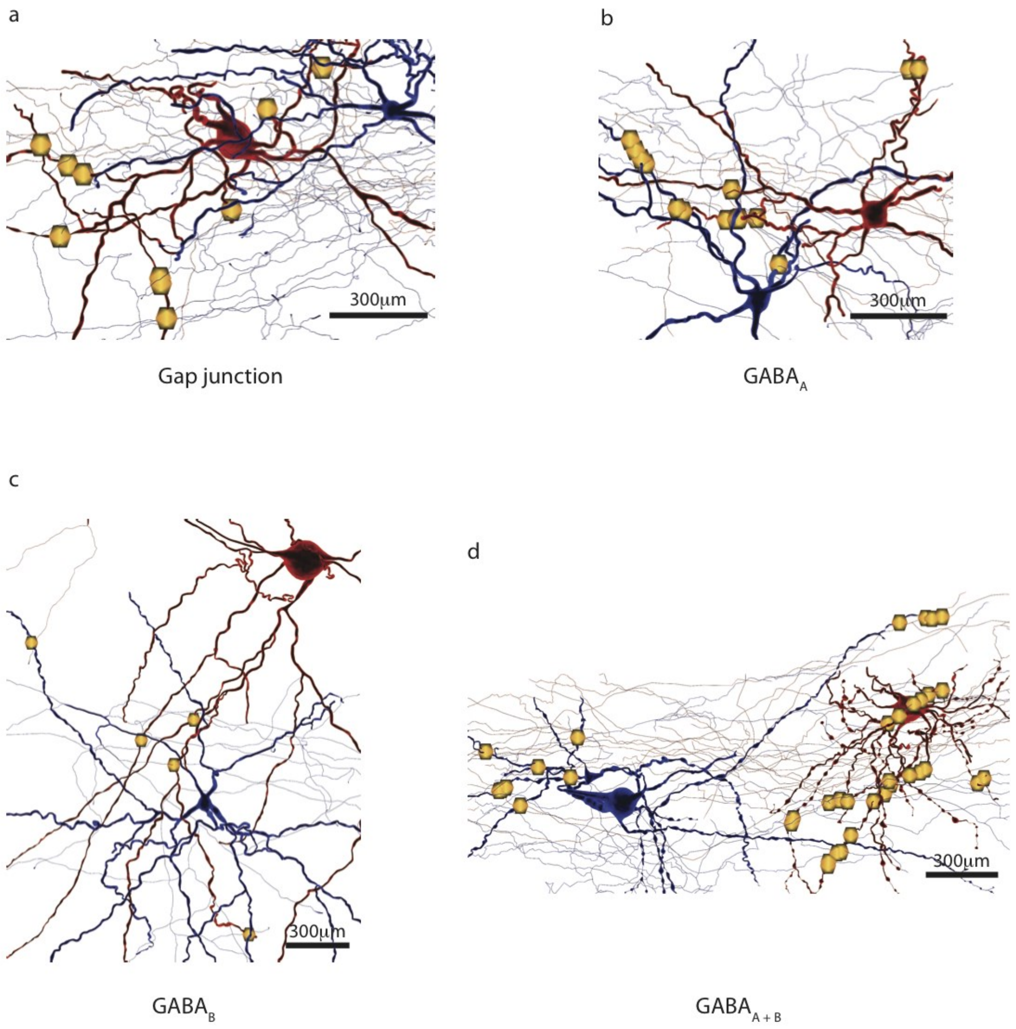

Supplementary Figure 7:

(a) is the close-up of a 3D rendering of the two cells with gap junctions and all the contact points marked with yellow hexagons. (b) for cells connected by $GABA_A$ (c) for cells connected by $GABA_B$ and (d) for cells with both $GABA_{A+B}$
